# Development of recommendations for a minimum dataset for Identifying Social factors that Stratify Health Opportunities and Outcomes (ISSHOOs) in pain research

**DOI:** 10.1016/j.mex.2023.102496

**Published:** 2023-11-24

**Authors:** Emma L. Karran, Aidan G. Cashin, Trevor Barker, Mark A. Boyd, Alessandro Chiarotto, Vina Mohabir, Jennifer Petkovic, Saurab Sharma, Peter Tugwell, G. Lorimer Moseley

**Affiliations:** a The ‘Identifying Social Factors that Stratify Health Opportunities and Outcomes (ISSHOOs) in Pain Research’ Collaboration; b Innovation, Implementation and Clinical Translation (IIMPACT) in Health, University of South Australia, Kaurna Country, Adelaide, South Australia, Australia; c Centre for Pain IMPACT, Neuroscience Research Australia, Sydney, New South Wales, Australia; d School of Health Sciences, University of New South Wales, Sydney, New South Wales, Australia; e Faculty of Health and Medical Sciences, University of Adelaide, Adelaide, South Australia, Australia; f Northern Adelaide Local health Network, Adelaide, South Australia, Australia; g Department of General Practice, Erasmus MC, University Medical Center, Rotterdam, the Netherlands; h Child Health Evaluative Sciences, Peter Gilgan Centre for Research and Learning, The Hospital for Sick Children, Toronto, Ontario, Canada; i Bruyere Research Institute, University of Ottawa, Ottawa, Canada; j Department of Medicine and School of Epidemiology, University of Ottawa, Ottawa, Canada

**Keywords:** Pain, Health equity, Social determinants of health, Protocol, Consensus methods

## Abstract

There is increasing recognition of the need for researchers to collect and report data that can illuminate health inequities. In pain research, routinely collecting equity-relevant data has the potential to inform about the generalisability of findings; whether the intervention has differential effects across strata of society; or it could be used to guide population targeting for clinical studies. Developing clarity and consensus on ***what*** data should be collected and ***how*** to collect it is required to prompt researchers to further consider equity issues in the planning, conduct, interpretation, and reporting of research. The overarching aim of the ‘Identifying Social Factors that Stratify Health Opportunities and Outcomes’ (ISSHOOs) in pain research project is to provide researchers in the pain field with recommendations to guide the routine collection of equity-relevant data. The design of this project is consistent with the methods outlined in the ‘Guidance for Developers of Health Research Reporting Guidelines’ and involves 4 stages: (i) Scoping review; (ii) Delphi Study; (iii) Consensus Meeting; and (iv) Focus Groups. This stakeholder-engaged project will produce a minimum dataset that has global, expert consensus. Results will be disseminated along with explanation and elaboration as a crucial step towards facilitating future action to address avoidable disparities in pain outcomes.

## Graphical abstract

A graphical abstract is attached with this submission.

**Figure:**
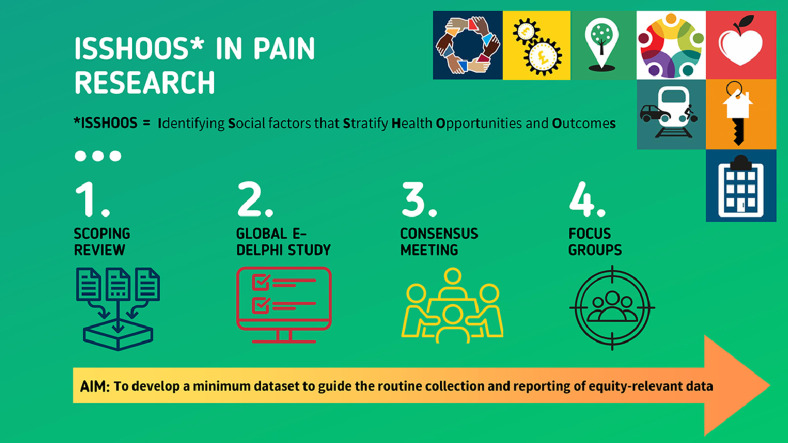

Specifications table

**Table:** 

Subject area:	Medicine and Dentistry
More specific subject area:	Pain
Name of your protocol:	Development of recommendations for a minimum dataset for Identifying Social factors that Stratify Health Opportunities and Outcomes (ISSHOOs) in pain research
Reagents/tools:	Not applicable
Experimental design:	Consensus Methods aligned with Moher D, Schulz KF, Simera I, Altman DG. Guidance for developers of health research reporting guidelines. *PLoS Medicine*. 2010;7(2):e1000217.
Trial registration:	Not applicable
Ethics:	We have obtained approval to conduct the Delphi study from the UniSA Human Research Ethics Committee (ID 204295). Further Ethics approval for project stages involving human participants will be confirmed before commencing participant recruitment.
Value of the protocol:	• A consensus approach to developing a minimum dataset to drive the routine collection of equity-relevant data in pain research is timely and important • This project has potential for broad application by researchers, journals, and funding organizations • The outputs of this project have the potential to facilitate future action to address pain-related health inequities

## Description of the protocol

### Project background and rationale

There is increasing recognition that health researchers must do better at collecting, reporting and interpreting ‘equity-relevant’ data [2], [3], [4]; a concern that is also drawing attention in the pain field [5], [6], [7], [8]. Knowing *what* data to collect and *how* to collect it can be challenging for researchers due to the complexity of issues, gaps in understanding and a lack of clear guidance. The development and implementation of clear, consensus-based recommendations is a crucial step towards improving equity considerations in pain research. This stakeholder-engaged consensus project is an important and timely initiative that has the potential to facilitate understanding of pain-related health inequalities and prompt action to address avoidable disparities in pain outcomes.

### Project aim

The overarching aim of ‘The ISSHOOs project’ is to provide all researchers in the pain field who recruit human participants with recommendations to guide the routine collection and reporting of equity-relevant data.

### Project objectives

The primary objective of this project is to develop a recommended minimum dataset to guide the collection of equity relevant data. The secondary objectives are to:

(i) Produce an explanation and elaboration document addressing: (a) why collection and reporting of this data is important; and (b) how this data can be used (i.e. suggestions for analysis and reporting); and
(ii) Consider and address the need to develop extended datasets for particular study types, contexts or populations; and/or guidance for adapting dataset items to suit the characteristics of the study population.

### Project design

The design of the ISSHOOs project is consistent with the methods outlined in the ‘Guidance for Developers of Health Research Reporting Guidelines’ [1]. This guidance can be readily applied to the current project and will produce tabulated recommendations and explanations to guide the collection, reporting and analysis of equity-relevant data.

The ISSHOOs in Pain Research Scoping Review protocol was registered on Open Science Framework (OSF) in June 2022. The Delphi Study protocol was registered on OSF in April 2023. Further detailed study protocols will be registered on OSF prior to commencing participant recruitment (as required).

### The ISSHOOs working group

The **Core Research Group (CRG)** is made up of the project leads (EK and LM) and eight contributing and advisory members (TB, MB, AGC, AC, VM, JP, SS, PT). The group includes international expertise in pain, equity, stakeholder engagement, consensus methods and lived experience of persistent pain. The CRG developed the protocol and will be responsible for the ongoing conduct and reporting of the ISSHOOs project. The CRG will establish a project ‘stakeholder and advisory group’ and assist with participant recruitment for the Delphi study.

The **Stakeholder and Advisory Group (STAG)** was established to provide expert oversight of the project. It involves 30 members from six continents and includes pain journal editors, representatives of pain associations and funding organizations, pain clinicians and people with a lived experience of chronic pain. STAG members are provided with the opportunity to contribute to study planning, provide oversight of study conduct, and will be invited to review and contribute suggestions related to the final research outputs. The STAG will also assist with participant recruitment for the project stages via their global networks.

### Study procedures

**Fig. 1:**
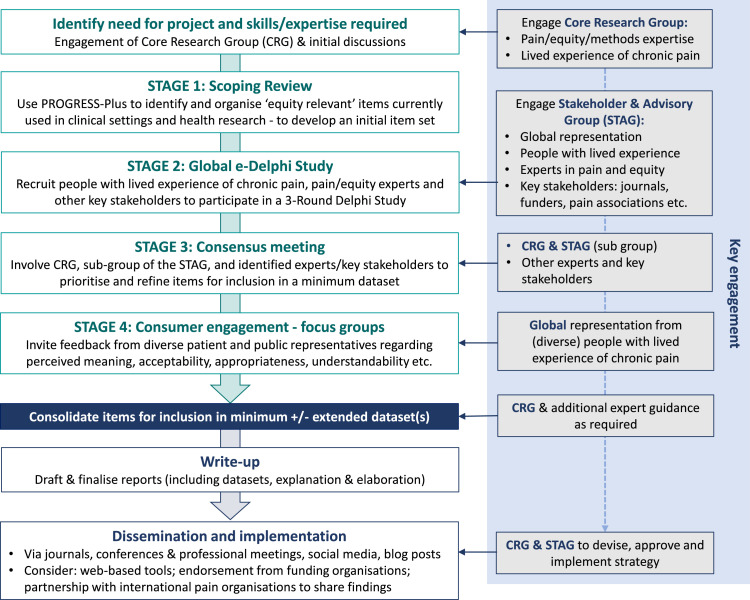
Overview of the ISSHOOs Project.

An overview of the project is provided in Fig. 1 and outlined in detail below.

STAGE 1: Scoping Review

The aim of the scoping review is to develop a comprehensive set of items (i.e., question and response sets) used to identify Social Determinants of Health (SDoH) (at the individual level) in clinical and/or research settings. These items will be mapped to the PROGRESS-Plus framework [9], [10], [11]. The specific research questions are:

(i) What items relevant to evaluating individual-level SDoH are included in social risk screening tools that have been developed to identify SDoH, social risk factors or social needs in clinical settings?
(ii) What screening items have been used to identify SDoH in equity-relevant health research?
(iii) Which of the PROGRESS-Plus characteristics do the items (from (i) and (ii) above) address?

In this scoping review we will apply ‘PROGRESS-Plus’ to identify relevant data, form a framework for data synthesis, and allow identification of any characteristics for which our scoping review retrieves minimal or no data. PROGRESS-Plus is an acronym used to identify characteristics that stratify health opportunities and outcomes and has been developed to facilitate the application of an ‘equity lens’ to the conduct, reporting, and use of research. PROGRESS refers to Place, Race and ethnicity, Occupation, Gender, Religion, Education, Socioeconomic status and Social capital. Plus refers to: (i) personal characteristics associated with discrimination (e.g. age, disability, sexual orientation); (ii) features of relationships (e.g. smoking parents, excluded from school; and (iii) time-dependent relationships (e.g. leaving the hospital, respite care, other instances where a person may be temporarily at a disadvantage).

Research Questions (i) and (ii) will be addressed by conducting two separate search stages, the results will be combined to address Research Question (iii). An (optional) third search stage will involve targeted searching if the data retrieved for any of the PROGRESS-Plus characteristics is inadequate.

### Data sources

A single researcher will systematically search the MEDLINE and Embase electronic databases to identify eligible studies.

### Search strategy: stage 1

Stage 1 of the scoping review will involve searching for *screening tools* that have been developed to identify the SDoH, social risks and/or social needs in clinical settings. The search strategy will contain several search terms for the two domains (SDoH; and screening) and will be limited to identify studies published since January 1, 2010. This date limitation avoids the identification of studies undertaken prior to the development a of contemporary awareness of health equity – informed by the 2008 PROGRESS Plus Framework [9,10], and the 2008 report of the WHO Commission on SDH [12]. Studies will be limited to those involving human participants; there will be no language restrictions. We will use English search terms to identify eligible studies.

The decision to include multi-domain screening tools identified by our search will be guided by the following criteria [13]: To be included, a tool needed to be designed for use in primary healthcare settings to ensure its relevance for informing clinical care or social intervention as opposed to other purposes (e.g., research). ‘Multi-domain’ was operationalized as a tool that included at least one social risk screening question in two or more of the following domains: economic stability (e.g., employment, income, and expenses), education (e.g., early childhood education, education level, and literacy), social and community context (e.g., social support systems, community engagement, and immigrant/refugee status), health and clinical care (e.g., access, coverage, and provider availability and cultural competence), neighborhood and physical environment (e.g., housing, transportation, and safety and crime), and food (e.g., food insecurity and access to healthy options).

### Search strategy: stage 2

Stage 2 of the scoping review will involve searching for ‘equity-relevant’ studies that have been published in 2021. Since equity-relevant studies focus on analyses across PROGRESS, these studies are expected to include individual *items* that have been used to identify SDoH. We will use a search strategy adapted from a published search filter devised to identify equity-relevant trials [14]. Firstly, we will conduct a search to identify individual- and cluster- randomized trials and longitudinal cohort studies. We will export the search results to Excel and randomly sort the studies. Two reviewers will then screen these studies for eligibility, beginning at the top of the (randomly sorted) list. The top 100 studies that meet our inclusion criteria will be included. Secondly, we will re-run the search strategy but replace the randomized trial design search terms for search terms that identify cross-sectional studies. Similarly, we will export the search results to Excel and randomly sort the studies, including the top 100 studies that meet our inclusion criteria. Including a total of 200 studies randomly selected from our search results is supported by the results of previous work indicating that including 200 ‘equity relevant’ studies provide broad (but incomplete) coverage across the PROGRESS-Plus characteristics [15]. Data will be extracted from the 200 included studies and the items used to identify the SDH will be mapped to the PROGRESS-Plus framework.

### Search strategy: stage 3

Stage 3 will only be undertaken if there are insufficient data items (i.e. <10 items) collected for any of the PROGRESS characteristics. This stage will involve separate ‘focused’ searches for the specific characteristic(s) as required. The search terms relating to the entire range of PROGRESS characteristics will be replaced by search terms for the characteristic of interest. Randomized and observational studies will be included. The search results will be exported to Excel and randomly sorted. Two reviewers will then screen these studies for eligibility, beginning at the top of the (randomly sorted) list. Studies will be included until we have extracted 10 data items relevant to each characteristic.

### Data management

For Search Strategy 1 we will export the articles identified in the database search into the *Covidence* systematic review management system (https://www.covidence.org/) where duplicates will be removed. Where English search terms yield study information in languages other than English, we will translate the title and abstract using Google Translate for title and abstract review, full-text review and data extraction. For Search Strategy 2 (and 3 - if required) we will export the results into Excel where they will be randomly sorted and screened. Any duplicates that are encountered will be removed.

### Study selection

Two reviewers will independently screen the titles and abstracts of all articles for eligibility according to the inclusion and exclusion criteria. The full text of potentially eligible studies will then be obtained, and the articles further screened for inclusion (with reasons for exclusion recorded). Any discrepancies or disagreements between the two reviewers will be discussed. If a disagreement is not resolved through this process a third reviewer will provide opinion and a majority decision will be made.

### Data extraction process

A single reviewer will extract the relevant data from included studies using a standardized and pilot-tested excel spreadsheet. A second reviewer will extract data from 10 % of the included studies from each of the search stages and the results will be cross-checked. Any inconsistencies will be resolved and corrected. A third reviewer will be consulted in the case of persisting disagreement. The following data will be extracted from all studies: study authors, year of publication, language of publication (if not English), country of study, study design, description of population and the PROGRESS-Plus-relevant screening items. Screening item details that will be extracted (as relevant) may include: the variable name (as reported – i.e. *what* was measured), *how* the variable was measured (i.e. question and response options and instructions when applicable) and any categories or cut-offs used. For the studies identified in stage 1, the following information will also be extracted: name of screening tool (if applicable), language of screening tool (if not English), validation of tool (Y/N – in current study or reported to have been previously carried out).

For the studies identified in stage 2: if detail concerning the precise wording of the PROGRESS-Plus-relevant question(s) is inadequate (in the published paper or online supplement) we will contact the study authors to request a copy of the questionnaire, or the specific items of interest in the questionnaire. Three emails will be sent (as required) over a 3-week period in an attempt to obtain this detail. In the case of non-response, the reviewers will match the (available) response options with an appropriate question.

### Data synthesis

The study characteristics will be summarized using descriptive statistics including means (and standard deviations) or medians (and range), and numbers (proportions). Each of the screening items identified in search stages 1–3 will be mapped to a PROGRESS-Plus characteristic and the results from all searches will be integrated as a final step. Results will be summarized using descriptive statistics such as frequencies and percentages and presented in tables and figures.

### Reporting

This review will be reported in accordance with the PRISMA extension for scoping reviews (PRISMA-ScR) [16] and submitted to a peer reviewed journal for publication. Stage 1 of the scoping review has been completed and published [17].

STAGE 2: Delphi Study

Our primary objective is to conduct a modified, e-Delphi study involving an international panel of experts and key stakeholders to assess the level of agreement on the importance of including items (question and response sets) in a minimum dataset, from an initial list. Our secondary objective is to identify the items that require modifying or clarifying (e.g. to be relevant to varied contexts or cultures) and elicit suggestions for refinement. Our methods will be guided by recommendations for Delphi methodology [18,19], and our study will be reported in accordance with the ACCORD (ACcurate COnsensus Reporting Document) guidelines [20].

### Participant eligibility, identification and recruitment

We will endeavor to involve representatives from all relevant expert and stakeholder groups in the study, according to the following definitions: *Experts* – Individuals with research expertise relevant to: (i) conducting primary research in the pain field (e.g. clinical trials, longitudinal cohort studies, cross-sectional studies), or (ii) one or more of the PROGRESS-Plus characteristics. To be classified as an ‘expert’ the researcher must have published 2 or more studies in the previous 5 years in the relevant field. *Stakeholders* – Individuals or representatives of groups/organizations who will be involved in the eventual endorsement, dissemination or use of the recommended dataset, or the potential to benefit from it. Stakeholders will include patients (people with lived experience of persistent pain); individuals associated with funding organizations, pain and pain advocacy associations, journal editors, policy makers and clinicians.

We aim to recruit 250–300 adult participants with representation from participation from patient, research and professional groups worldwide. While we require a high level of expertise in the panel, we also value the insights and perspectives of a diverse range of participants with varied backgrounds and experience. To be eligible, the experts and stakeholders will need to confirm that they have the time available to participate and are willing to be involved in all components of the study. Potential participants will be identified using the following strategies:

a. Each of the ISSHOOs STAG members will be requested to personally contact 5–10 people from their networks via email, to notify them of the Delphi study and suggest that they consider being involved. The STAG members will provide study information (a pro-forma will be provided to the STAG members to disseminate) and will request that the recipients contact the ISSHOOs lead investigator (ELK) to express their interest in being involved.
b. The CRG will devise a list of potential participants from their own networks, with deliberate consideration of achieving breadth of expertise across the PROGRESS-Plus characteristics. As above, the CRG members will provide study information and request that the recipients contact the ISSHOOs lead investigator (ELK) to express their interest in being involved.
c. Literature scoping. Where expertise relating to one or more PROGRESS-Plus characteristics is lacking we will scope the literature to identify researchers who have recently (i.e. during the past five years) published two or more original research papers in relevant fields. We will access publicly available email addresses to provide these researchers with study information and request that they reply to indicate their interest in being involved. The CRG will also consider stakeholder groups that would be ideally represented in this study (and are not), and the lead investigator will make contact.

### Participant consent

Potential participants will contact the lead investigator (EK) via email to express their interest in being involved in the study. ELK will reply to ask that they provide some brief information about their relevant expertise, their preferred language for the Delphi study (if not English) and confirm their availability to complete all 3 rounds of the Delphi survey. A Participant Information Sheet will be emailed, and individuals will be offered the opportunity to discuss the study and their participation (if required). Participants will be given a further opportunity to read the information sheet when they receive Round 1 of the Delphi study and will confirm their consent to be involved by commencing the study.

### Generation of the initial item list

Consistent with a modified Delphi process, we will generate the initial list of items for the Round 1 Delphi survey through a scoping review and expert consultation. An initial item list, defined by PROGRESS-Plus, will be developed via the scoping review described above. If the CRG determines that this is inadequate or requires modification, they will undertake expert consultation to expand or adapt the item list.

### Procedure

We will use REDCap software to deliver a series of three electronic surveys and receive responses. We estimate that the first round will take 30–40 min to complete. Rounds 2 and 3 will take approximately 30 min to complete. Panellists will be requested to respond to questions to collect demographic information and provide details about their relevant expertise prior to participating in Round 1. Rounds 1 and 2 will involve presenting the participants with lists of items to evaluate the social determinants of health. These items will be organized according to the PROGRESS-Plus framework with separate sections for each PROGRESS-Plus category. For each of the 3 rounds, panel members will be given 3 weeks to respond to the questionnaire. This timeframe has been recommended to maintain panel interest and to minimise study attrition. There will be a 6 to 8-week time interval between each round.

### Round 1 survey development and piloting

Two surveys will be developed – one designed for completion by patient/public participants (people with a lived experience of persistent pain) (Survey A); the other designed for completion by researchers, clinicians or other professionals (Survey B). Both surveys will contain identical items (for rating) but will have differing explanatory information. Each will be piloted by 3 experts (minimum) who will be asked to provide feedback related to usability, readability and relevance. Feedback from the pilot process will be collated and the surveys will be edited/revised accordingly.

### Data collection – Round 1

A link to commence the Delphi process will be sent via email to panel participants. Participants will be given the opportunity to re-read the information sheet and they will confirm their consent by commencing the survey. Participants will be asked to provide demographic details and respond to questions asking about their professional background and/or expertise relevant to this study. They will then be provided with the initial item set. Participants will be asked to allocate a response on a 9-point Likert scale to rate how important they believe it is that each item be included in a minimum dataset. The Likert scale will be anchored with ‘not important’ and ‘extremely important’.

We will apply the RAND/UCLA (Research ANd Development/University of California Los Angeles) appropriateness method to set thresholds for disagreement and agreement on importance for inclusion of items minimum dataset [21]. (This method is detailed in data analysis section). Items which reach our pre-defined criteria for agreement will be excluded from the Round 2 survey.

### Data collection - Round 2

Panel members who completed Round 1 will be emailed an electronic link to the Round 2 survey. This will include all (non-excluded) items and any additional items (suggested by participants in Round 1). Participants will be provided summary of the collated responses from Round 1 (for each item carried over into Round 2) for both participant groups (i.e. Group A: people with a lived experience of persistent pain, and Group B: ‘other’ experts/stakeholders). Participants will also be sent a record of their own Round 1 scores and will be asked to consider the aggregated panel medians (from both groups) when re-scoring the importance of each item. Newly nominated items from Round 1 will also be presented for rating. In addition to item ratings of importance, participants will be asked (for each item): “Do you think this question needs refining for your context/a global context” (yes/no). Panellists will be instructed that items with a mean score of ≤3 are considered not important and will be excluded from the minimum dataset and items with a mean score of ≥7 are considered important and will be included in the minimum dataset. Panel members will also be asked to contribute additional items for consideration in subsequent rounds. Responses from round 2 will be collated and analyzed; items which reach agreement will be removed from the Round 3 survey.

### Data collection - Round 3

Panel members who completed Round 2 will be emailed an electronic link to the Round 3 questionnaire. This questionnaire will contain:

a. Items for which agreement has not been reached (i.e. the ‘uncertain’ items only). The results of Round 2 will again be distributed back to the panellists (for these items) to allow them to see where their response stands in relation to Groups A and B. In Round 3, panel members will be asked to vote to ‘include’ or ‘exclude’ the items. Participants will also be given the opportunity to provide open-ended responses to explain their vote and/or provide suggestions for how the question could be re-worded or refined.
b. Items to which participants to the question of: “Do you think this question needs refining for your context/a global context” with ‘yes’. Participants will be given the opportunity to provide open-ended responses with suggestions for how the question could be re-worded or refined.

### Confidentiality and data storage

Study data will be collected and stored on REDCap - a secure web application for building and managing online surveys and databases. All anonymised data will be subsequently extracted by the lead investigator (ELK) to password protected files that will be securely stored on a secure server at the University of South Australia. In accordance with the University of South Australia's Ownership and Retention of Data Policy and the Australian Code for the Conduct of Research all data will be securely stored for a minimum of 5 years.

### Analysis

Quantitative data will be analyzed using IBM SPSS v28 (IMB Corp, Armonk, NY, USA). Descriptive statistics will be presented for the demographic characteristics of panel members, survey response rates, and withdrawals. Round 1 free-text responses suggesting additional items will be collated and considered by the CRG for inclusion. Similar suggestions may be amalgamated, and the need for minor wording refinements will be considered. The free text comments from Round 3 will be synthesized by the research team to identify the key concerns of the panellists. These concerns and suggestions will be presented for discussion at the consensus meeting (Stage 4).

### Quantitative analysis of scoring

We will analyse the responses from each round using the RAND/UCLA appropriateness method which we will modify to ask panellists to rate “importance” (rather than “appropriateness”). This method considers the median panel rating (for each item) and its dispersion to provide an index of importance and agreement [21]. This involves calculating the median score, the interpercentile range (IPR) (30th and 70th) and the interpercentile range adjusted for symmetry (IPRAS) for each item being rated. We will consider agreement to be present when the IPR ≤ IPRAS and disagreement to be present when the IPR ≥ IPRAS for a given item. Analysis will be undertaken for Groups A and B independently. For the analysis of Rounds 1 and 2, items considered for the minimum dataset will be categorized following the RAND/UCLA definitions:

• “Include”: panel median of 7–9, without disagreement (i.e. IPR ≤ IPRAS))
• “Uncertain”: panel median of 4–6, or any median with disagreement (i.e. IPR ≤ IPRAS)
• “Exclude”: panel median of 1–3, without disagreement (i.e. IPR ≤ IPRAS)

Items rated as “uncertain” will proceed to the subsequent round.

For the analysis of Round 3, consensus will be set as follows:

• ≥60 % vote “include”: include item
• 41 %–59 % vote “include”: uncertain
• ≤40 % vote “exclude”: exclude item

Clear discrepancies between the stakeholder groups (A and B) and items rated as ‘uncertain’ will be discussed at the subsequent Consensus Meeting.

### Minimising attrition

To minimise drop-out, we will send personalized reminder emails to panellists identified as ‘non-completers’ at 7, 14 and 18 days from the initial mailing. At the final reminder we will instruct participants that they have 3 days remaining to complete the survey. Participants will be advised that if they do not complete the survey in this timeframe their results will not be analyzed, and they will not be invited to participate in subsequent rounds. We will highlight the importance of completing all 3 survey rounds but will emphasise that all participation in this study is voluntary.

STAGE 3: Consensus Meeting

The list of agreed items produced by the Delphi study will inform an online consensus meeting to consolidate and prioritise key items to be included in the minimum dataset. The consensus meeting will involve the CRG, a subgroup of members from the STAG and invited members with additional expertise (if required). It will include people with a lived experience of persistent pain. A questionnaire will be developed to collect demographic data from Consensus Meeting participants that broadly captures the PROGRESS-Plus characteristics; also position (academic or other) and relevant expertise. A meeting schedule will be devised that aims to reach consensus regarding (i) the recommended minimum dataset, (ii) any extended datasets required, (iii) concepts requiring explanation and/or elaboration, (iv) agreement on who will draft the final publication or series of publications (e.g. a core writing team who will send the documents to the wider consensus team for feedback), and (v) a dissemination strategy.

STAGE 4: Focus Groups

Stage 4 will involve online, constructed focus group with adults from the general public and people with a lived experience of persistent pain. Targeted recruitment will occur for these groups via the global networks of the CRG. We will conduct three or four focus groups, each involving four to eight participants. Since we aim to include representatives from all continents (except Antarctica) the focus groups will be organized and scheduled to suit the time zones of the participants. The members of the focus groups will be presented with the recommended minimum dataset and a discussion will be led to explore aspects such as perceived meaning, acceptability, appropriateness, understandability. The results of the focus groups will be integrated (by the CRG) to produce a revised version of the dataset– this version will be sent back to the focus groups for further (individual) feedback prior to consolidation if items. We will aim to involve a diverse range of people – of varied ages, socio-economic status, education level and cultural backgrounds.

Writing and dissemination

Completion of this project will involve the drafting and production of recommendations including specification of a recommended minimum dataset; explanation and elaboration of the rationale for collecting this data; and suggestions for how it can be used. Attention will be given to ensuring clear and precise reporting that is also suitable for all relevant stakeholders. The STAG and members of the Consensus Meeting will be invited to review and provide feedback on the draft document. A dissemination strategy will be developed by the CRG, with guidance and approval from the STAG. We will disseminate the final recommendations via journals, conferences, and professional meetings across multiple disciplines. We will aim to publish the outputs in one or more leading pain (and/or medical) journals, with an accompanying social media strategy. We will also liaise with relevant journal editors, international pain associations and funding agencies to consider potential for endorsement and collaborative strategies to promote uptake. We will make the minimum dataset and accompanying outputs available on the open ISSHOOs Project web domain (www.isshoos.org) alongside audio-visual guides and tools to facilitate data collection and reporting. Ongoing work will seek to involve multi-lingual experts in back-translation processes to translate the minimum dataset and accompanying resources.

### Publication plan

• Publication 1: Study Protocol
• Publication 2: Scoping Review Stage 1
• Publication 3: Scoping Review Stage 2
• Publication 4: Delphi Study
• Publication 5: Expert Engagement and Refining (Consensus Meeting and Consumer Focus Groups)
• Publications 6 & 7: Simultaneous publications for the minimum dataset and the ‘explanation and elaboration’ papers

## Additional information

An overview of the ISSHOOs Project and research team, project information, and links to published (open access) protocols and manuscripts can be found at www.isshoos.org. Updates to this protocol (and the protocols of the individual studies in this project) are available on the ISSHOOs website and on OSF.

## CRediT authorship contribution statement

**Emma L. Karran:** Conceptualization, Methodology, Investigation, Writing – original draft, Project administration. **Aidan G. Cashin:** Conceptualization, Methodology, Investigation, Writing – review & editing. **Trevor Barker:** Conceptualization, Investigation, Writing – review & editing. **Mark A. Boyd:** Conceptualization, Investigation, Writing – review & editing. **Alessandro Chiarotto:** Conceptualization, Methodology, Investigation, Writing – review & editing. **Vina Mohabir:** Conceptualization, Methodology, Investigation, Writing – review & editing. **Jennifer Petkovic:** Conceptualization, Methodology, Writing – review & editing. **Saurab Sharma:** Conceptualization, Methodology, Investigation, Writing – review & editing. **Peter Tugwell:** Conceptualization, Methodology, Writing – review & editing. **G. Lorimer Moseley:** Conceptualization, Methodology, Writing – review & editing, Funding acquisition.

## Declaration of Competing Interest

The authors declare the following financial interests/personal relationships which may be considered as potential competing interests:

ELK has received speaker fees for lectures on pain and rehabilitation from professional and scientific bodies, and reimbursement of travel costs related to presentations at scientific conferences/symposia. GLM has received support from: Reality Health, ConnectHealth UK, Institutes of Health California, AIA Australia, Workers’ Compensation Boards and professional sporting organizations in Australia, Europe, South and North America. Professional and scientific bodies have reimbursed him for travel costs related to presentation of research on pain and pain education at scientific conferences/symposia. He has received speaker fees for lectures on pain, pain education and rehabilitation and conference travel support from Sequirus. He receives royalties for books on pain and pain education. PT has received consulting fees to provide independent medical consultation and professional services. He is an independent Committee Member for clinical trial Data Safety Monitoring Boards for FDA approved trials being conducted by UCB Biopharma GmbH & SPRL, Parexel International, Prahealth Sciences. PT is an [unpaid] Chair of the Management Subcommittee of the Executive Committee of a registered non-profit independent medical research organization, OMERACT. OMERACT receives unrestricted educational grants from the American College of Rheumatology, European League of Rheumatology and several pharmaceutical companies listed in this section, which is used to support fellows, international patient groups and support a major international bi-annual conference which results in many peer-reviewed publications. There are no competing interests for any other author.

## Data Availability

No data was used for the research described in the article.
